# Retrospective research: identifying and conducting phylogenetic analyses on four Orf virus strains isolated in Yunnan province between 2021 and 2023—revealing their significance and characteristic features

**DOI:** 10.3389/fvets.2024.1481809

**Published:** 2024-12-03

**Authors:** Jiarui Xie, Meiling Kou, Yinan Wang, Xiaohang Su, Huafeng Gao, Haisheng Miao

**Affiliations:** Yunnan Tropical and Subtropical Animal Virus Disease Laboratory, Yunnan Academy of Animal Husbandry and Veterinary Sciences, Kunming, Yunnan, China

**Keywords:** Contagious Eczema, Orf virus, ORFV011, ORFV059, phylogenetic analysis

## Abstract

Contagious Eczema (CE), caused by ORFV, impacts sheep and goats globally, with severe symptoms and economic losses. The ORFV situation in Yunnan, China, was unclear before 2021–2023 study. Eleven scab samples from goats on small farms in three Yunnan municipalities were collected. Four ORFV strains were isolated and characterized using scanning electron microscopy, cytopathic effect observation, and PCR. Phylogenetic analyses of ORFV011 and ORFV059 genes showed significant results. For ORFV011, the nucleotide similarity of the four strains to D1701 strain was 98.4–99%. For ORFV059, it was 97.2–97.9% with OV-SA00 strain. These findings suggest gene rearrangements and interactions among strains during Yunnan’s ORFV outbreak, forming a unique evolutionary lineage. Our study is the first comprehensive one on Yunnan’s ORFV prevalence with in-depth phylogenetic analysis. It has important implications. In vaccine development, understanding genetic variances helps create better vaccines. For disease control, customized strategies like targeted quarantine and disinfection can be designed based on strain characteristics. From a public health aspect, as CE is zoonotic, closely monitoring ORFV in goats aids in predicting and preventing human infections, thus being significant for protecting goats against CE in Yunnan.

## 1 Introduction

Contagious Ecthyma (CE), a disease of substantial significance within the domains of veterinary and public health, is induced by the Orf virus (ORFV). This highly contagious and rapidly proliferating illness predominantly afflicts small ruminants such as sheep, goats, cattle, camels, and wildlife (1, 2). The principal manifestations of CE are typified by the emergence of thickened cutaneous lesions on diverse body parts, encompassing the eyelids, lips, oral mucosa, around the nasal region, and even on the nipples of goats and sheep. Notably, CE presents a grave zoonotic hazard, particularly for individuals who come into direct contact with infected animals, potentially resulting in human infections and subsequent health complications (3, 4).

Although CE generally manifests a high incidence of the disease with a relatively low fatality rate, it exerts a particularly severe impact on juvenile animals. The mortality rate among juveniles is frequently elevated, presumably due to their incapacity to nurse effectively on account of the painful lesions inflicted by the virus (5). The survival capabilities of ORFV further exacerbate the situation. Its epitheliotropic and resilient nature, along with its immune evasion mechanisms, empower it to endure for months within conducive environments and elude clearance by the host immune system (6, 7). Consequently, the prevention and treatment of CE remain arduous tasks, with no currently available highly efficacious approaches (8). The transmission of this disease typically occurs through direct contact with an infected entity, often via damaged skin or abrasions on the lips, nostrils, and mouth caused by dried feeds or plants (9). The diagnosis of CE usually entails observing clinical symptoms and deploying various laboratory methodologies such as electron microscopy, virus isolation, and molecular assays to identify the presence of ORFV (10).

ORFV, belonging to the genus Parapoxvirus within the family Poxviridae, is categorized as the smallest member of the subfamily Chordopoxvirinae. It exhibits a global distribution, with a particular prevalence in countries that are major producers of sheep and goat (11, 12). The virus harbors a linear double-stranded DNA genome, estimated to span from 134 to 139 kb in size (13, 14). Analogous to other poxviruses, its genome comprises a core region and variable genomic termini (15). The pivotal genes situated in the central region, specifically ORFV009 to ORFV111, are highly conserved and play indispensable roles in replication, transcription, and virion assembly (13). These genes are commonly exploited for ORFV detection, marker development, and epidemiology analyses (16).

In recent years, the study of ORFV has attracted escalating attention due to its profound impact on animal health and the potential ramifications for human health. Polymerase chain reaction (PCR) amplification, sequencing, and phylogenetic analyses have been extensively utilized to explore the diversity of ORFV genes in numerous countries (17, 18). Comprehending the evolutionary origins and relevant molecular epidemiological data of ORFV is of paramount importance for effectively curbing its spread and prevalence.

Firstly, delving into the evolutionary origins of ORFV enables scientists to discern the genetic variations and transmission pathways of the virus. This knowledge imparts crucial insights into its adaptability across different hosts and environmental conditions. For instance, recent studies have demonstrated that ORFV can acclimate to different host species and environmental alterations, which may precipitate changes in its pathogenicity and transmissibility (1, 19–21). Such understanding is fundamental for formulating rational prevention strategies, developing effective vaccines, and evaluating their efficacy.

Secondly, molecular epidemiological data, including the genomic sequences of the virus, its variation characteristics, and transmission networks, can unveil the prevalence characteristics and trends of ORFV in different regions. This information is not only vital for epidemic surveillance but also assists public health agencies to identify potential outbreak sources and high-risk areas. For example, a recent study in a specific region illustrated that analyzing molecular epidemiological data of ORFV led to the early detection of an emerging outbreak and the implementation of timely control measures (19, 22–25).

Finally, a comprehensive analysis integrating evolutionary origins and molecular epidemiology enables the formulation of more precise and targeted control measures. This could involve optimizing vaccination strategies based on the specific genetic characteristics of local strains and intensifying surveillance in high-risk areas. Recent research has emphasized the importance of customizing vaccination strategies to local ORFV strains to improve vaccine efficacy (22, 23, 26, 27). Ultimately, these efforts are aimed at effectively controlling ORFV and mitigating its threats to both animal and human health.

Multiple studies have also attested to the significance of the primary envelope proteins of ORFV in eliciting an immune response (28). For example, the ORFV011 protein, a key immunogenic protein, can stimulate a strong antibody response by activating lymphocytes from draining lymph nodes (29). Similarly, the ORFV059 protein shows potential as a possible antigen in subunit vaccines targeting antigenically similar ORFV strains. It is also reported to be involved in the generation of neutralizing antibodies within the host and plays a significant role in the viral life cycle (30). Vaccination has thus emerged as an effective means of preventing ORFV infection (31). However, it should be noted that genetic alterations in prevalent strains under current immune pressures may reduce the efficacy of inactivated and attenuated vaccines, potentially leaving the host vulnerable to reinfection (32). To ensure the effectiveness of vaccination, it is essential to have detailed information about local pandemic strains. Additionally, attenuated vaccines carry the risk of virulence reversion and viral spread (32).

Previous studies have documented cross-species and transregional transmission of ORFV (27), as well as the co-prevalence of multiple strains (33). In recent years, cases of ORFV infection and outbreaks have been reported in several countries (27) and various regions of China, including Northwest, Northeast, East, Southern, and Central China (18), resulting in substantial economic losses to the sheep and goat breeding industry and posing a threat to human health worldwide (2). However, despite the widespread occurrence of ORFV in different parts of the world and China, the prevalence of ORFV in Yunnan Province has remained unreported. This dearth of information poses a significant quandary as Yunnan Province is a region with a rich diversity of livestock, including sheep and goats. The presence of ORFV in this region could have a profound impact on the local livestock industry, potentially leading to economic losses similar to those seen in other regions. Moreover, given the zoonotic nature of CE, the unreported prevalence of ORFV in Yunnan Province also raises concerns about potential human infections in the area.

Therefore, our study endeavors to bridge this critical knowledge gap by procuring comprehensive epidemiological information about ORFV and CE in Yunnan Province. Specifically, we collected scab samples from goats on small farms suspected of experiencing CE outbreaks from 2021 to 2023 in Yunnan Province. We then isolated ORFV from these samples and analyzed their ORFV011 and ORFV059 genes. The novelty of our study lies in providing the first meticulous exploration of the prevalence and genetic characteristics of ORFV in the Yunnan Province region. This research will contribute significantly to the prevention of ORFV infection and outbreaks in the area, thereby safeguarding the health of both animals and humans. By understanding the specific genetic makeup and prevalence of ORFV in Yunnan Province, we can develop more targeted prevention strategies, such as tailored vaccination programs and enhanced surveillance measures, which will be crucial for protecting the local livestock industry and reducing the potential zoonotic risks to human health.

In conclusion, our study is not only aimed at unearthing the epidemiological situation of ORFV in Yunnan Province but also at providing valuable insights for formulating more effective prevention and control measures, which will have a profound impact on protecting the health of animals and humans in the region.

In this section, we have incorporated several recent studies (1, 19–27) to fortify the context and relevance of our research. The ever-evolving nature of viral epidemiology demands that we keep abreast of the latest research findings to better understand the problem at hand and develop more effective solutions.

## 2 Materials and methods

### 2.1 Sample collection

Scab samples were collected from the papules of goats on farms suspected of having CE outbreaks in various regions of Yunnan Province (China) between 2021 and 2023. Tissue specimens were collected by scraping crusts from affected areas, particularly form the lips and nostrils of goats exhibiting typical pathological changes associated with Orf infection. In total, 11 samples, each sample weighs approximately 15 grams, were obtained and stored in 15 mL centrifuge tubes. These samples were kept at a temperature of 2–8°C in Virus Sample Stabilization Solution (Beyotime, Shanghai, China) and promptly transported to the Yunnan Tropical and Subtropical Animal Virus Disease Laboratory as soon as possible for further analysis. Detailed information about the collected samples is shown in Table 1.

**Table 1:** Detailed information about the collected samples.

Sample label	Sampling time	Sampling location	Geographic information		Sampling object	Gender of livestock species	Month of age	Sampling site	Sample volume	Treatment
			Longitude and latitude	Altitude						
1	Dec-2021	Shilin County, Yunnan Province	24°77′44.76″N, 103°28′84.70″E	1,682 m	Black goat	♂	1-month-old	Scabs and skin lesions around mouth and nose	1 sample, approximately 15 g, kept in 1 centrifuge tube (15 mL)	Stored at 2–8°C in Virus Sample Stabilization Solution transported to laboratory as soon as possible
2	Dec-2021	Shilin County, Yunnan Province	24°77′44.76”N, 103°28′84.70″E	1,682 m	Black goat	♂	2-month-old	Scabs and skin lesions around mouth and nose	1 sample, approximately 15 g, kept in 1 centrifuge tube (15 mL)	Stored at 2–8°C in Virus Sample Stabilization Solution transported to laboratory as soon as possible
3	Dec-2021	Shilin County, Yunnan Province	24°77′44.76″N, 103°28′84.70″E	1,682 m	Black goat	♀	1-month-old	Scabs and skin lesions around mouth and nose	1 sample, approximately 15 g, kept in 1 centrifuge tube (15 mL)	Stored at 2–8°C in Virus Sample Stabilization Solution transported to laboratory as soon as possible
7	Dec-2022	Yiliang County, Yunnan Province	24°91′97.63″N, 103°14′04.80″E	1,537 m	Boer goat	♀	3-month-old	Scabs and skin lesions around mouth and nose	1 sample, approximately 15 g, kept in 1 centrifuge tube (15 mL)	Stored at 2–8°C in Virus Sample Stabilization Solution transported to laboratory as soon as possible
8	Dec-2022	Yiliang County, Yunnan Province	24°91′97.63″N, 103°14′04.80″E	1,537 m	Boer goat	♀	1-month-old	Scabs and skin lesions around mouth and nose	1 sample, approximately 15 g, kept in 1 centrifuge tube (15 mL)	Stored at 2–8°C in Virus Sample Stabilization Solution transported to laboratory as soon as possible
9	Dec-2022	Yiliang County, Yunnan Province	24°91′97.63″N, 103°14′04.80″E	1,537 m	Boer goat	♂	1-month-old	Scabs and skin lesions around mouth and nose	1 sample, approximately 15 g, kept in 1 centrifuge tube (15 mL)	Stored at 2–8°C in Virus Sample Stabilization Solution transported to laboratory as soon as possible
4	Jul-2023	Shilin County, Yunnan Province	24°77′44.76″N, 103°28′84.70″E	1,682 m	Black goat	♂	2-month-old	Scabs and skin lesions around mouth and nose	1 sample, approximately 15 g, kept in 1 centrifuge tube (15 mL)	Stored at 2–8°C in Virus Sample Stabilization Solution transported to laboratory as soon as possible
5	Jul-2023	Shilin County, Yunnan Province	24°77′44.76″N, 103°28′84.70″E	1,682 m	Black goat	♂	1-month-old	Scabs and skin lesions around mouth and nose	1 sample, approximately 15 g, kept in 1 centrifuge tube (15 mL)	Stored at 2–8°C in Virus Sample Stabilization Solution transported to laboratory as soon as possible
6	Jul-2023	Shilin County, Yunnan Province	24°77′44.76″N, 103°28′84.70″E	1,682 m	Black goat	♀	1-month-old	Scabs and skin lesions around mouth and nose	1 sample, approximately 15 g, kept in 1 centrifuge tube (15 mL)	Stored at 2–8°C in Virus Sample Stabilization Solution transported to laboratory as soon as possible
10	May-2023	Unity Township, Kunming, Yunnan Province	25°07′16.06″N, 102°53′40.84″E	2,358 m	Black goat	♀	2-month-old	Scabs and skin lesions around mouth and nose	1 sample, approximately 15 g, kept in 1 centrifuge tube (15 mL)	Stored at 2–8°C in Virus Sample Stabilization Solution transported to laboratory as soon as possible
11	May-2023	Unity Township, Kunming, Yunnan Province	25°07′16.06″N, 102°53′40.84″E	2,358 m	Black goat	♀	2-month-old	Scabs and skin lesions around mouth and nose	1 sample, approximately 15 g, kept in 1 centrifuge tube (15 mL)	Stored at 2–8°C in Virus Sample Stabilization Solution transported to laboratory as soon as possible

### 2.2 Goat testis cell isolation and culture

A testis was collected from a newborn goat at an abattoir and thoroughly washed with pre-warmed 1 × phosphate-buffer saline (PBS; Servicebio, Wuhan, China). After removing the covering cuticle, the testis was fragmented using an ophthalmology scissor and digested with 0.05% trypsin–EDTA (Gibco, Waltham, MA, USA) for 3–5 min. The digestion was then stopped by adding twice the volume of Dulbecco’s Modified Minimal Essential Medium (DMEM; Gibco, Waltham, MA, USA) containing 10% fetal bovine serum (FBS; Gibco, Waltham, MA, USA) and 1% penicillin and streptomycin (PS, Solarbio, Beijing, China). The resulting suspension was filtered through a sterile muslin cloth. After three rounds of digestion, and final suspension was centrifuged at 1,000 rpm for 10 min. The resulting cell pellet was resuspended in DMEM containing 10% FBS and 1% PS and cultured at 37°C in a CO_2_ incubator for subsequent experiments.

### 2.3 Identification and isolation of ORFV

The collected scab samples were ground into powder using a quartz milling device and prepared as a 10% suspension in DMEM containing 1% PS. The suspension was then filtered through a 0.45 μm syringe filter (Millipore, Burlington, MA, USA). When the goat testis cells reached approximate 80% confluence, an aliquot of the suspension was added to the flask to infect them for 2 h. After the infection period, the goat testis cells were washed twice with PBS, and DMEM with 10% FBS and 1% PS was added for 72 h of further cultivation. Morphologies of these testis cells were observed and captured daily under a microscope. When approximately 80–90% of the cells exhibited CPE, repeated freezing and thawing was performed, and then the cell debris was removed by centrifugation at 1,500×*g* for 5 min at 37°C. The cell supernatant was collected, ultra-centrifuged using 35% sucrose solution, centrifuged at 32,000×*g* for 4 h, the supernatant was poured off, and the bottom wall of the centrifuge tube was rinsed repeatedly with PBS to collect the concentrated viral solution. Electron microscopy (Hitachi, Tokyo, Japan) was used to stain the supernatant containing mature virus particles with 2% phosphotungstic acid.

Testis cells, both infected and uninfected, were harvested, and their genomic DNA was extracted using a Tianamp Genomic DNA Kit (Tiangen, Beijing, China). Simultaneously, total DNA from an ORFV freeze-dried vaccine purchased from Shandong Huahong Biological Engineering Co., (Shandong, China) was also extracted. The concentrations of these extracted DNA were measured using a Nanodrop 2000 spectrophotometer and used as templates for subsequent PCR amplification analyses. The *ORFV011* and *ORFV059* gene fragments in each sample were amplified using the primers in shown in Table 2 (18). The PCR amplification assay was conducted in a 50 μL reaction system containing 100 ng template DNA, primers, and 2 × PrimeSTAR® Max Premix (Takara, Shiga, Japan). The PCR amplification conditions for ORFV011 (B2L) gene was as follows: initial pre-denaturation at 95°C for 5 min, followed by 30 cycles of denaturation at 95°C for 30 s, annealing at 62°C for 30 s, extension at 72°C for 70 s, and final extension cycle at 72°C for 10 min; and for ORFV059 (F1L): 30 cycles of denaturation at 95°C for 30 s, annealing at 56°C for 30 s, and extension at 72°C for 70 s, final extension for 10 min at 72°C. The PCR products were separated by 1% agarose gel electrophoresis with Gold View I (Solarbio, Beijing, China) for 40 min at 100 V. The amplified products that exhibited electrophoretic bands in the correct positions were sent to Tsingke Biotechnology Co., Ltd. (Kunming, China) for gene sequencing. Bi-directional primers were used to determine the full-length sequences of the ORFV 011 and ORFV 059 genes. Each gene was sequenced three times, and the repetitive gene sequences were compared to remove or correct misidentified bases. The sequences were assembled and edited using MEGA6 software.

**Table 2:** Sequences of primers used in the PCR amplification assay.

Gene	Primer sequences	Tm	Length of product
*ORFV011*	F: 5′-ATGTGGCCGTTCTCCTCCATCC-3′	54.1°C	1,137 bp
	R: 5′-TTAATTTATTGGTTTGCAGAACT-3′		
*ORFV059*	F: 5′-TCACACGATGGCCGTGACCAGCAGC-3′	54°C	1,029 bp
	R: 5′-ATGGATCCACCCGAAATCACGGCCT′3′		

### 2.4 Phylogenetic analyses

After sequencing (DNA Sequencer: ABI3730), the raw sequence data of the *ORFV011* and *ORFV059* genes isolated from our samples were assembled using the Staden package.^1^ Multiple alignments was performed using the MUSCLE condon algorithm implemented in MEGA software (v6). To construct a phylogenetic tree, we retrieved 19 *ORFV011* gene sequences and 22 *ORFV059* gene sequences from GenBank. Detailed information about these sequences is shown in Table 3. Briefly, sequences of our isolated *ORFV011* and *ORFV059* gene segments were compared with these retrieved sequences using the Basic Local Alignment Search Tool (BLAST)^2^ with default settings. In the process of selecting sequences for phylogenetic tree analysis and construction, the following key factors are primarily taken into account: the diversity of species is a key factor in the selection of sequences for phylogenetic tree analysis and construction. The selection of species or strains with disparate evolutionary histories enables a more accurate delineation of their evolutionary relationships. By comparing the differences and similarities among the sequences, it is possible to identify common ancestors and evolutionary pathways. The selection of genes is a crucial aspect of phylogenetic analysis. The selection of specific gene sequences can facilitate the generation of more reliable data for phylogenetic analysis. The quality of the data is of great importance. It is essential to ensure that the selected sequences have been sequenced with high-quality data, are complete, and have minimal gaps in order to guarantee the accuracy of the analysis. Similarity and variability are two key factors that must be considered when analyzing genetic sequences. The selection of sequences exhibiting appropriate similarity and variability among the target species is essential for the effective differentiation of these species and the construction of a reasonable phylogenetic tree. The selected sequences must be accessible in public databases, facilitating alignment and the acquisition of additional reference data. Evolutionary Models: It is essential to consider whether the selected sequences are suitable for specific evolutionary model analysis, as different sequences may be appropriate for different models. The phylogenetic analysis was performed using the Tamura-Nei model, and the confidence intervals were estimated by a bootstrap algorithm applying 1,000 iterations. Bootstrap values (%) of ≥50% are displayed at the tree branch nodes. The scale bar indicates branch length. Molecular phylogeny and genetic relatedness of these isolated ORFV strains with other ORFV strains were calculated using percentage similarity and pairwise distances. Gene sequence comparison and analysis of variation at the nucleotide and amino acid levels were conducted using the MegAlign program in DNA Star software.

**Table 3:** Detailed information about the retrieved genes from GenBank for phylogenetic analysis.

No.	GenBank accession number	Country	Year of collection	Specie	Gene
1	KU976392/NX	Hunan, China	2015	Goat	*ORFV011*
2	KP010356/SJ1	Fujian, China	2012	Goat	*ORFV011*
3	ON380500/Mukteswar-vaccine-passage50	India	2005	Goat	*ORFV011*
4	ON380499/Mukteswar-passage9	India	2005	Goat	*ORFV011*
5	OP279270/UPM01	Malaysia	2020	Goat	*ORFV011*
6	PP805861/FJ-2403	Fujian, China	2024	Goat	*ORFV011*
7	MW537048/UPM/HSN-20	Malaysia	2018	Goat	*ORFV011*/*ORFV059*
8	MN648219/CL18	China	2018	Sheep	*ORFV011*
9	AY386263/OV-IA82	USA	1982	Sheep	*ORFV011*/*ORFV059*
10	JN088051/NE2	Brazil	1993	Goat	*ORFV011*
11	HM133903/D1701	Germany		Sheep	*ORFV011*/*ORFV059*
12	AY386264/OV-SA00	USA		Homo-sapiens	*ORFV011*/*ORFV059*
13	KT438521/ATARC/001	Ethiopia	2008	Sheep	*ORFV011*
14	MN422326/IRFH18	Iran	2019	Goat	*ORFV011*
15	MT272780/UDUS	Nigeria	2019	Goat	*ORFV011*
16	LR594616/IHUMI-1	France		Cutaneous biopsy	*ORFV011*
17	KF837136/B029	Germany	1996	Homo-sapiens	*ORFV011*/*ORFV059*
18	DQ184476/NZ2	New-Zealand			*ORFV011*/*ORFV059*
19	KU976387/LYJ	Hunan, China	2015	Goat	*ORFV011*
20	KF234407	Guangzhou, China	2011	Sheep	*ORFV059*
21	ON805832/NAV	Spain	2018	Sheep	*ORFV059*
23	KC569751/OV-HN3	China	2012	Sheep	*ORFV059*
24	KF703748/Xinjiang	China	2013	Goat	*ORFV059*
25	KP010353/YX	Fujian, China	2012	Goat	*ORFV059*
26	KY412871/Meghalaya	India	2003	Goat	*ORFV059*
27	MF489125/AH1404	Anhui, China	2014	Goat	*ORFV059*
28	MF489129/AH1610	Anhui, China	2016	Goat	*ORFV059*
29	MF489130/AH1612	Anhui, China	2016	Goat	*ORFV059*
30	MF489132/AH1704	Anhui, China	2017	Goat	*ORFV059*
32	ON805833/ARA	Spain	2018	Sheep	*ORFV059*
33	OQ455254/TR-ORF-Izolat-2003-ORFV059	Turkey	2003	Goat	*ORFV059*
34	OQ455255/TR-ORF-2019.2-ORFV059	Turkey	2019	Sheep	*ORFV059*
35	MG712417/SY17	Jilin, China	2016	Sheep	*ORFV059*
36	FJ808075/Jilin	Jilin, China	2008	Sheep	*ORFV059*
37	PP805864/FJ-2403	Fujian, China	2024	Goat	*ORFV059*

### 2.5 Protein structure and physicochemical properties analyses

The amino acid composition and physicochemical properties of the *ORFV011* and *ORFV059* proteins were analyzed using the Expasy ProtParam tool.^3^ Additionally, SOPMA software^4^ was used to analyze and predict the secondary structure of the proteins encoded by our isolated *ORFV011* and *ORFV059* genes.

## 3 Results

### 3.1 PCR amplification of *ORFV011* and *ORFV059* genes

**Figure 1:**
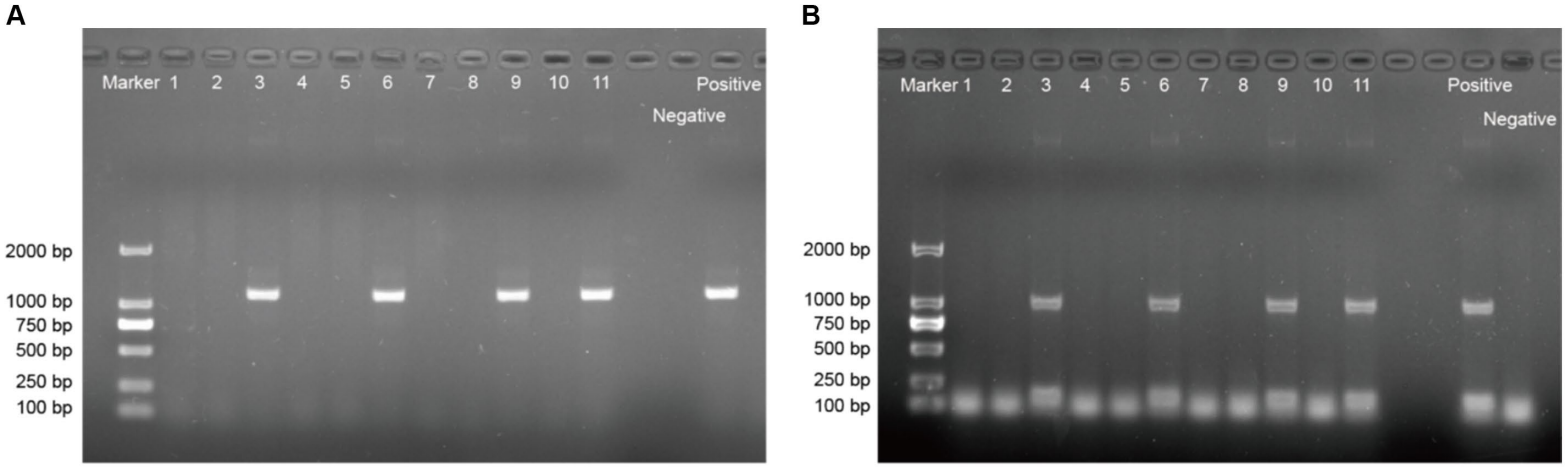
Gel images showing the bands of ORFV011 gene **(A)** and ORFV059 gene **(B)** in PCR products from each sample. 1–11: Labels of samples in Table 1. The positive control DNA was the total DNA extracted from a freeze-dried Orf virus (ORFV) vaccine purchased from Shandong Huahong Biological Engineering Co., Ltd. (Shandong, China). The negative control was nuclease-free sterile water.

From 2021 to 2023, a total of 11 scab samples were collected form goats on farms suspected of having CE breakouts. The PCR products of 4 of these samples, labeled as ORFV/goat/YNSLi/China/2021/Yunnan, ORFV/goat/YNSLi/China/2023/Yunnan, ORFV/goat/YNYLn/China/2022/Yunnan, and ORFV/goat/YNTJe/China/2023/Yunnan, exhibited obvious bands at the positions of 1,137 bp and 1,029 bp (Figure 1). Table 4 provides detailed information about these 4 ORFV stains and their corresponding scab samples. The nucleotide sequences generated in this study were deposited in the NCBI GenBank sequence database with accession numbers PP733995–PP733998 for the *ORFV011* gene and PP733999–PP734002 for the *ORFV059* gene (Table 4).

**Table 4:** Detailed information about the isolated ORFV strains.

ORFV strains	Location	Geographic information		Collection time	Breed	History/clinical symptoms	Sample type	GenBank accession number	
		Longitude and latitude	Altitude					*ORFV011*	*ORFV059*
YNSLi/China/2021/Yunnan	Shilin County, Yunnan Province	24°77′44.76″N, 103°28′84.70″E	1,682 m	2021	Black goat	Scabs and skin lesions around mouth and nose	Skin scab	PP733996	PP734000
YNSLi/China/2023/Yunnan	Shilin County, Yunnan Province	24°77′44.76″N, 103°28′84.70″E	1,682 m	2023	Black goat	Scabs and skin lesions around mouth and nose	Skin scab	PP733997	PP734001
YNYLn/China/2022/Yunnan	Yiliang County, Yunnan Province	24°91′97.63″N, 103°14′04.80″E	1,537 m	2022	Boer goat	Scabs and skin lesions around mouth and nose	Skin scab	PP733995	PP733999
YNTJe/China/2023/Yunnan	Unity Township, Kunming, Yunnan Province	25°07′16.06″N, 102°53′40.84″E	2,358 m	2023	Black Goat	Scabs and skin Lesions around mouth and nose	Skin Scab	PP733998	PP734002

### 3.2 Cytopathic effects of isolated ORFV strains on goat testis cells

**Figure 2:**
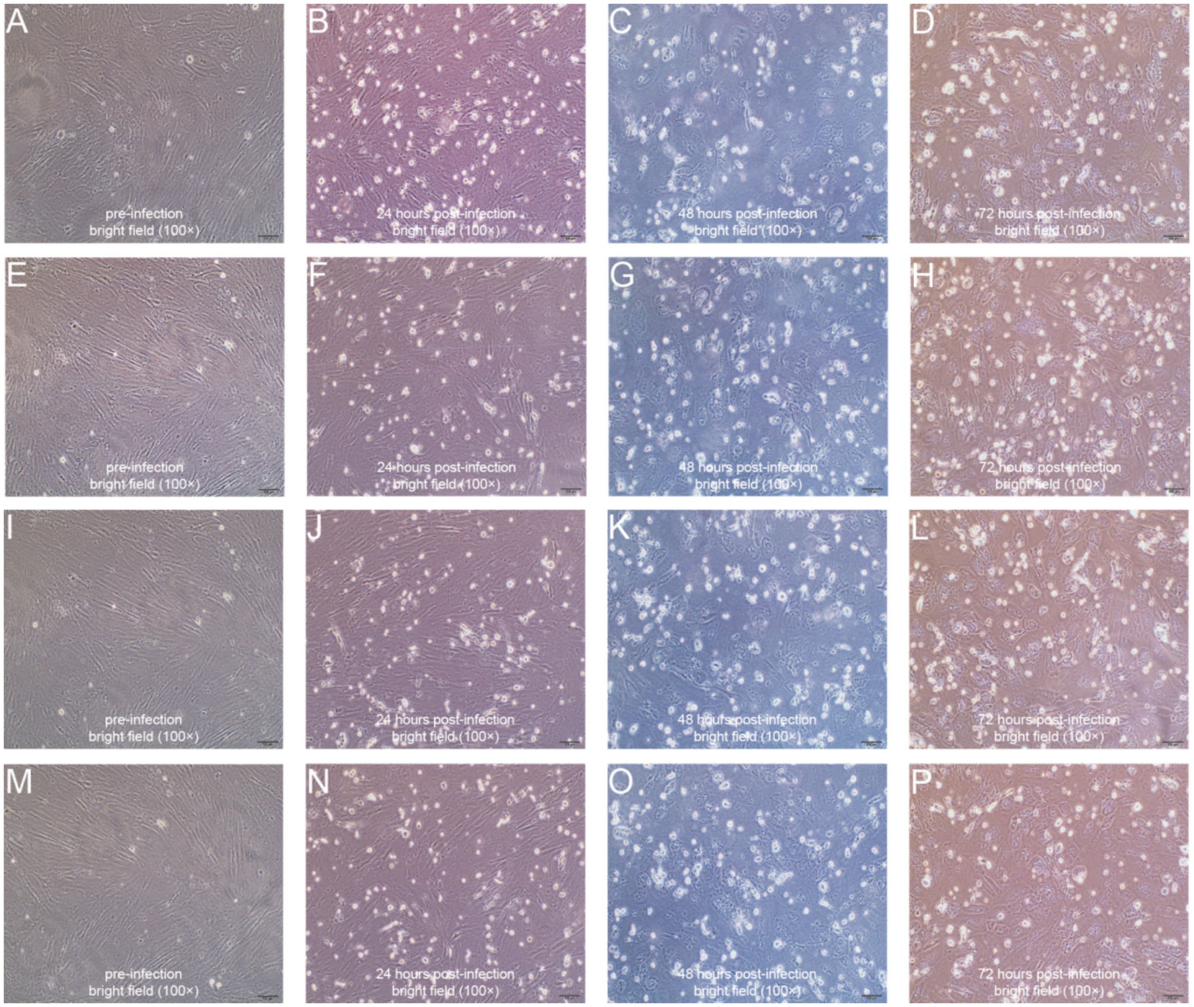
Representative microscopy images illustrating the morphologies of primary goat testis cells. **(A–D)** YNSLi/China/2021/Yunnan cells before **(A)**, 24 h post- **(B)**, 48 h post- **(C)**, and 72 h post- **(D)** infection; **(E–H)** YNSLi/China/2023/Yunnan cells before **(E)**, 24 h post- **(F)**, 48 h post- **(G)**, and 72 h post- **(H)** infection; **(I–L)** YNYLn/China/2022/Yunnan cells before **(I)**, 24 h post- **(J)**, 48 h post- **(K)**, and 72 h post- **(L)** infection; **(M–P)** YNTJe/China/2023/Yunnan before **(M)**, 24 h post- **(N)**, 48 h post- **(O)**, and 72 h post- **(P)** infection.

To further validate the virulence of our isolated ORFV strains, we infected primary goat testis cells with these four ORFV strains. Before infection, the testis cells exhibited a spindle-shaped or elongated appearance with prominent, elongated nuclei, suggesting a fibroblast-like morphology typical of connective tissue cells (Figures 2A,E,I,M). At 24 h post-infection, the cells became round and began to detach, cell density and confluence decreased, and increased cellular debris and irregular intercellular spaces were observed (Figures 2B,F,J,N). At 48 h post-infection, the testis cells exhibited extensive cytopathic effects, including increased cell rounding, further reduced cell density, more pronounced cellular debris, and greater disruption of intercellular organization (Figures 2C,G,K,O). At 72 h post-infection, the testis cells exhibited severe cytopathic effects, including significant cell detachment, extensive cell rounding, high levels of cellular debris, and a marked decrease in overall cell density (Figures 2D,H,L,P).

### 3.3 Morphological characteristics of the isolated ORFV stains

**Figure 3:**
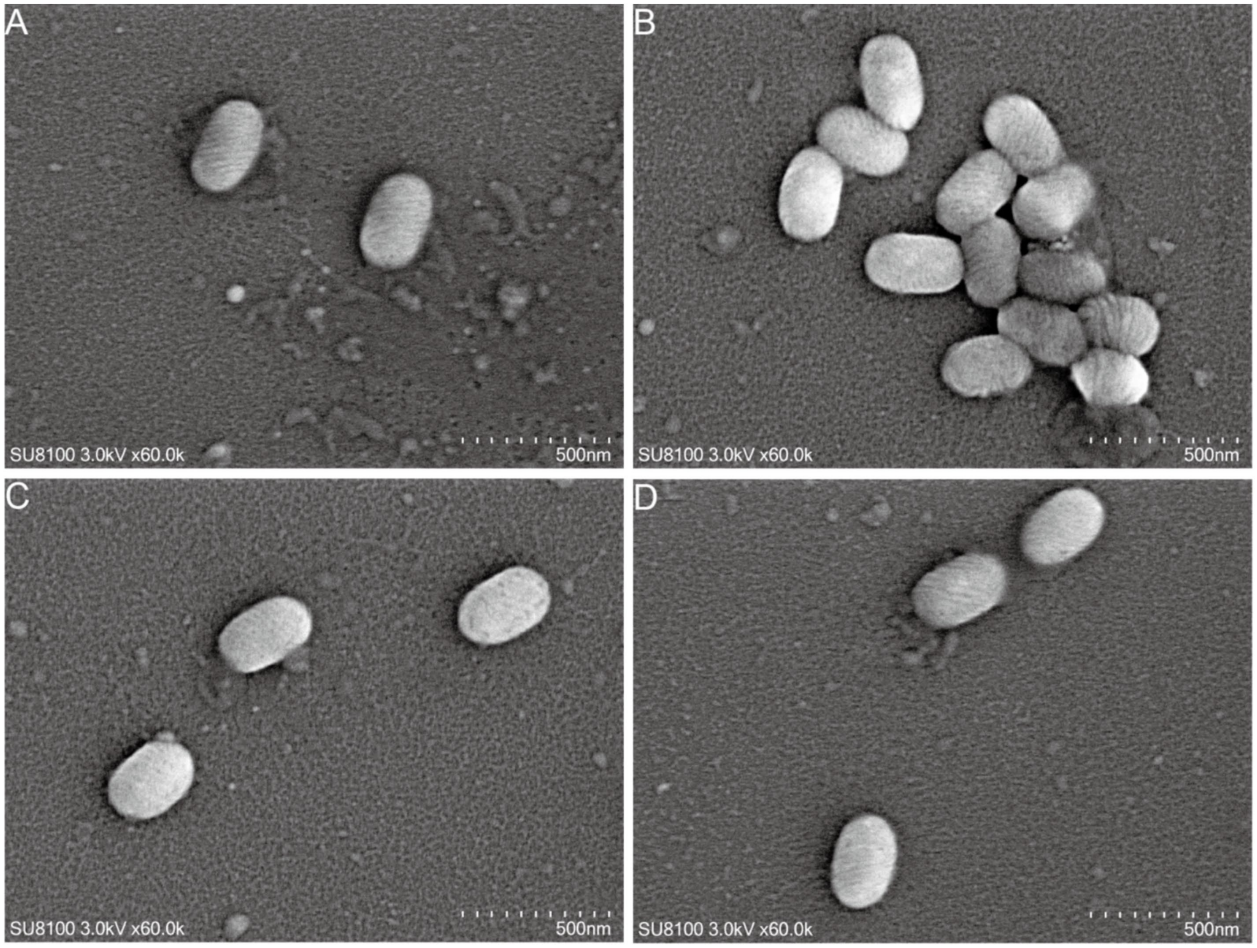
Electron microscopic examination of the ORFV strain ORFV/goat/YNSLi/China/2021/Yunnan **(A)**, ORFV/goat/YNSLi/China/2023/Yunnan **(B)**, ORFV/goat/YNYLn/China/2022/Yunnan **(C)**, and ORFV/goat/YNTJe/China/2023/Yunnan **(D)** from infected primary goat testis cells.

We also observed the morphological characteristics of the isolated ORFV strains using TEM. As shown in Figure 3, the viral particles presented a typical elliptical pile-like structure with a helical cross pattern and appeared rope-like in cross sections. We observed viral particles of length ~260 nm and width ~180 nm (Scale bar = 500 nm).

### 3.4 Homology analysis of the isolated ORFV strains

**Figure 4:**
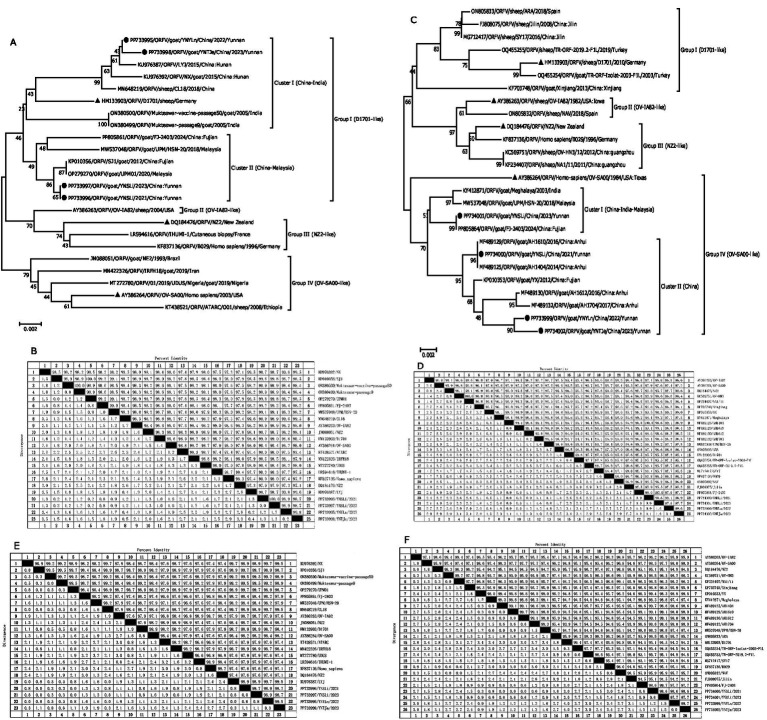
Phylogenetic analyses of the ORFV011 **(A)** and ORFV059 **(C)** genes of our isolated ORFV strains, along with the corresponding reference strains, as well as the nucleotide sequence distances of the ORFV011 **(B)** and ORFV059 **(D)** genes, the amino acid sequence distances of the ORFV011 **(E)** and ORFV059 **(F)** genes. Black circulars represent the strains isolated in the present study, while black triangles represent the reference strains. The nucleotide sequences were aligned using the Clustal W method. The phylogenetic tree was constructed using the MEGA 6.0 software based on the Kimura-2 parameter model to evaluate the evolutionary relationships among different strains. A neighbor-joining algorithm with 1,000 bootstrap replicates was used. Bootstrap values (%) of ≥50% are displayed at the tree branch nodes. The scale bar indicates branch length.

To determine the population characteristics of our isolated ORFV strains, we conducted phylogenetic and biogeographic analyses of the *ORFV011* and *ORFV059* genes. The genetic lineage of each gene predominantly consisted of Asian strains, and as shown in Figures 4A,C, all these *ORFV011* and *ORFV059* sequences were mainly concentrated in 4 groups: SA00-like (the earliest known SA00 strain with a complete genome), NZ2-like (the earliest known NZ2 strain with a complete genome), D1701-like (the earliest known D1701 strain with a complete -genome), and OV-IA82-like (the earliest known OV-IA82 strain with a complete genome) (25).

Molecular phylogeny and genetic relatedness of the isolated ORFV strains with the 19 retrieved ORFV strains were calculated using percentage similarity and pairwise distances. The results of the *ORFV011* gene sequence comparison showed that all 4 isolated ORFV stains belonged to Group I (D1701-like). The percentage similarity observed of the *ORFV011* gene with the 19 reference strains ranged from 97.1 to 99.8% for nucleotides and from 97.4 to 99.7% for amino acid sequences (Figures 4B,E and Table 5). The nucleotide and amino acid sequence similarities with the D1701 strain ranged from 98.4 to 99% and from 98.4 to 98.9%, respectively, while those with the NZ2 strain ranged from 97.1 to 97.9% and 97.1 to 97.6%, respectively (Figures 4B,E and Table 5). The results of *ORFV059* gene sequence comparison showed that all four isolated ORFV stains belonged to Group IV (OV-SA00-like). The percentage similarity of the *ORFV059* gene with the 22 reference strains ranged from 95.1 to 100% for nucleotides and 95.1 to 99.7% for amino acid sequences (Figures 4D,F and Table 6). The nucleotide and amino acid sequence similarities with the D1701 strain ranged from 95.1 to 95.7% and from 94.2 to 95.1%, respectively, while those with the NZ2 strain ranged from 95.6 to 96% and from 94.8 to 95.1%, respectively (Figures 4D,F and Table 6). The phylogenetic tree analysis reveals that for the four strains isolated in Yunnan, the higher the bootstrap values of the evolutionary branches they form (usually considered to be ≥50% or ≥70%, etc.), the more reliable the branch structure is. This also implies that the genetic distance relationships among the strains reflected by this branch are more trustworthy.

**Table 5:** Summary of the highest nucleotide and amino acid sequence identities of the *ORFV011* genes from our isolated ORFV strains.

Strains	Maximum nucleotide sequence identity	Maximum amino acid sequence identity
YNSLi/2021	99.8%:	99.7%:
	OP279270/ORFV/goat/UPM01/Malaysia/2020	OP279270/ORFV/goat/UPM01/Malaysia/2020
	KP010356/0RFV/SJ1/goat/2012/China: Fujian	KP010356/0RFV/SJ1/goat/2012/China: Fujian
YNSLi/ 2023	99.8%:	99.7%:
	OP279270/ORFV/goat/UPM01/Malaysia/2020	OP279270/ORFV/goat/UPM01/Malaysia/2020
	KP010356/0RFV/SJ1/goat/2012/China: Fujian	KP010356/0RFV/SJ1/goat/2012/China: Fujian
	100%:	YNSLi/2021
	YNSLi/2021	
YNTJe/ 2023	99.6%:	99.5%:
	KU976387/ORFV/LYJ/2015/China: Hunan	KU976387/ORFV/LYJ/2015/China: Hunan
	99.8%:	YNYLn/2022
	YNYLn/2022	
YNYLn/2022	99.8%:	99.7%:
	KU976387/ORFV/LYJ/2015/China: Hunan	KU976387/ORFV/LYJ/2015/China: Hunan

**Table 6:** Summary of the highest nucleotide and amino acid sequence identities of the *ORFV059* genes from our isolated ORFV strains.

Strains	Maximum nucleotide sequence identity	Maximum amino acid sequence identity
YNSLi/2021	100%:	99.7%:
	MF489125/ORFV/goat/AH1404/2014/China: Anhui	MF489125/ORFV/goat/AH1404/2014/China: Anhui
YNSLi/2023	99.2%:	98.8%:
	KY412871/ORFV/goat/Meghalaya/2003/India	KY412871/ORFV/goat/Meghalaya/2003/India
	MW537048/ORFV/goat/UPM/HSN-20/2018/Malaysia	MW537048/ORFV/goat/UPM/HSN-20/2018/Malaysia
	100%:	99.7%:
	PP805864/ORFV/goat/FJ-2403/2024/China: Fujian	PP805864/ORFV/goat/FJ-2403/2024/China: Fujian
YNTJe/2023	99.6%:	99.7%:
	YNYLn/2022	YNYLn/2022
	99%:	98.8%:
	KP010353/ORFV/goat/YX/2012/China: Fujian	KP010353/ORFV/goat/YX/2012/China: Fujian
YNYLn/2022	99.2%:	98.8%:
	KP010353/ORFV/goat/YX/2012/China: Fujian	KP010353/ORFV/goat/YX/2012/China: Fujian

### 3.5 Analyses of protein physicochemical properties and secondary structures

Overall, the physicochemical properties of the *ORFV011* and *ORFV059* proteins from the 4 isolated ORFV stains differed slightly in terms of amino acid composition, molecular weight, theoretical pI, negative and positive residues, half-life, mean hydropathy, aliphathy, and instability compared to the NZ2 and D1701 strains. Specifically, the half-life of amino acids encoded by the *ORFV011* gene in mammalian reticulocytes was 4.4 h. The aliphatic index of proteins encoded by the *ORFV011* gene in the 4 isolated strains ranged from 18.47 to 18.82. The *ORFV011* proteins in our isolated ORFV stains had a hydropathy index of 0.948–0.951 and an instability factor of 63.94–64.43, while the *ORFV011* proteins in the NZ2 and D1701 strains had a hydrophobicity index of 0.947 and 0.945 and an instability factor of 64.57 and 63.60, respectively. The hydrophobicity index and the instability coefficient of the Yunnan strains were found to be rather moderate (Table 7). The half-life of amino acids encoded by the *ORFV059* gene in mammalian reticulocytes was also 4.4 h. The aliphatic index of proteins encoded by the *ORFV059* gene in our isolated ORFV strains ranged from 17.3 to 17.69. The *ORFV059* proteins in our isolated ORFV strains had a hydropathy index of 0.972–0.979 and an instability factor of 61.13–61.63, while the *ORFV059* proteins in the NZ2 and D1701 strains had a hydrophobicity index of 0.981 and 0.971 and an instability factor of 61.18 and 61.08, respectively (Table 8). The protein secondary structure of *ORFV011* and *ORFV059* proteins from the isolated ORFV strains also differed slightly compared to the NZ2 and D1701 strains. Detailed information about the structures of these proteins is shown in Tables 9, 10.

**Table 7:** Primary structures and physicochemical properties of the protein encoded by the *ORFV011* gene.

Strains	Amino acid composition	Molecular weight	Theoretical pI	Negative residues	Positive residues	Instability	Half-life	Aliphathy	Grand average of hydropathicity
ORF/YNSLi/CHA/2021	379	94,175.67	4.96	0	0	63.94	4.4 h	18.47	0.951
ORF/YNSLi/CHA/2023	379	94,175.67	4.96	0	0	63.94	4.4 h	18.47	0.951
ORF/YNTJe/CHA/2023	379	94,179.59	4.96	0	0	64.25	4.4 h	18.82	0.948
ORF/YNYLn/CHA/2022	379	94,179.59	4.96	0	0	64.43	4.4 h	18.82	0.948
NZ2	379	94,267.70	4.96	0	0	64.57	4.4 h	18.82	0.947
D1701	379	94,215.66	4.96	0	0	63.60	4.4 h	18.47	0.945

**Table 8:** Primary structures and physicochemical properties of the protein encoded by the *ORFV059* gene.

Strains	Amino acid composition	Molecular weight	Theoretical pI	Negative residues	Positive residues	Instability	Half-life	Aliphathy	Grand average of hydropathicity
ORF/YNSLi/CHA/2021	345	85,538.23	4.97	0	0	61.40	4.4 h	17.3	0.979
ORF/YNSLi/CHA/2023	345	85,584.17	4.97	0	0	61.63	4.4 h	17.69	0.972
ORF/YNTJe/CHA/2023	345	85,584.17	4.97	0	0	61.34	4.4 h	17.69	0.972
ORF/YNYLn/CHA/2022	345	85,588.23	4.97	0	0	61.13	4.4 h	17.69	0.978
NZ2	345	84,010.44	4.97	0	0	61.18	4.4 h	17.61	0.981
D1701	345	85,045.51	4.97	0	0	61.08	4.4 h	17.61	0.971

**Table 9:** Predictive of the secondary structures of *ORFV011* proteins.

Strains	α-helix (%)	β-folding (%)	Irregularly coiled (%)	Extended chain (%)
ORF/YNSLi/CHA/2021	30.16	5.03	41.53	23.28
ORF/YNSLi/CHA/2023	30.16	5.03	41.53	23.28
ORF/YNTJe/CHA/2023	31.75	5.56	39.42	23.28
ORF/YNYLn/CHA/2022	30.95	5.03	40.48	23.54
NZ2	31.75	4.50	40.74	23.02
D1701	32.80	4.76	39.95	22.49

**Table 10:** Predictive of the secondary structures of *ORFV059* proteins.

Strains	α-helix (%)	β-folding (%)	Irregularly coiled (%)	Extended chain (%)
ORF/YNSLi/CHA/2021	39.71	5.29	38.53	16.47
ORF/YNSLi/CHA/2023	40.59	5.59	35.29	18.53
ORF/YNTJe/CHA/2023	40.59	5.59	35.29	18.53
ORF/YNYLn/CHA/2022	40.59	4.71	35.88	18.82
NZ2	41.92	5.09	36.23	16.77
D1701	41.72	4.73	36.39	17.16

## 4 Discussion

### 4.1 Overview of ORFV outbreaks: implications and future considerations

From 1975 to 2023, the occurrence of ORFV outbreaks across multiple countries worldwide demonstrated a global epidemic distribution pattern. Notably, the period between 2011 and 2018 witnessed the highest epidemiological intensity, with India and China being severely affected. The majority of the viral population is composed of Asian strains, which can be categorized into four major groups: the SA00-like group, the IA82-like group, the D1701-like group, and the NZ2-like group. The IA82-like group stands out for its high virulence and significant geographic heterogeneity, while the SA00-like group predominantly consists of Asian strains (25). The genetic analyses conducted on the ORFV011 and ORFV059 genes of the isolated ORFV strains provide valuable insights into the population characteristics of these strains. However, it is essential to place these findings within the broader context of Orf virus (ORFV) epidemiology and control strategies to fully understand their implications. The fact that the genetic lineage of each gene predominantly consisted of Asian strains. This has significant implications for understanding the spread and evolution of ORFV in Asia. For instance, it may suggest that there have been historical patterns of transmission and adaptation within this region that have led to the prevalence of these particular genetic groups. In the context of epidemiology, knowledge of the genetic relatedness of the isolated strains can help in tracing the possible sources of infection and predicting the potential spread patterns. For example, the strains isolated in Yunnan show a close genetic relationship to strains from other regions within Asia, it could imply that there have been recent or past movements of infected animals or vectors that have facilitated the spread of ORFV across different areas. This information is crucial for implementing effective surveillance and control measures, such as targeted monitoring of areas with a high likelihood of virus introduction or spread, and for developing region-specific prevention strategies.

The manifestation of lip lesions in goats on affected farms led to the request for researchers to conduct molecular diagnostics for lip herpes. This was crucial in an attempt to prevent the escalation of epidemics. However, several factors contributed to the restricted sample collection. Firstly, the varying levels of participation from different farming households and operations meant that not all were equally forthcoming in providing samples. Some relevant personnel also expressed concerns regarding large-scale sampling efforts, perhaps due to potential disruptions to their normal farming activities or concerns about the impact on their livestock. Additionally, to minimize the risk of transmitting other potential pathogens, conscious efforts were made to avoid sampling in densely populated areas of the farms. Moreover, the inherent randomness within the experimental procedures during the virus isolation process further limited the isolation to only 4 distinct viral strains.

Future Research Implications: Understanding the specific factors that led to the peak in epidemiological intensity during 2011–2018 is of great importance. This could involve in-depth investigations into changes in environmental conditions (such as temperature, humidity, and rainfall patterns), animal husbandry practices (including feeding regimens, housing conditions, and vaccination schedules), and market dynamics (like the movement and trading of livestock across different regions). By identifying these factors, we can better predict future outbreaks and develop more targeted prevention strategies. Exploring the relationship between the virulence of the IA82-like group and its genetic makeup could provide valuable insights. Future studies could focus on identifying specific genes or genetic mutations within this group that contribute to its high virulence. This knowledge could then be used to develop novel prevention strategies or therapeutics that target these specific genetic elements.

Practical Application Implications: The need for molecular diagnostics due to lip lesions emphasizes the importance of early detection in epidemic control. Future efforts should focus on developing more rapid, accurate, and accessible molecular diagnostic tools that can be easily implemented on farms. This could involve the development of portable diagnostic kits or the integration of new technologies such as point-of-care testing. The challenges faced in sample collection highlight the necessity for improved sampling protocols. Future research could explore alternative sampling methods that are not only more comprehensive but also less invasive and less likely to introduce additional risks. For example, non-invasive sampling techniques such as saliva or fecal sampling could be investigated, along with methods to ensure the reliability and accuracy of results from such samples.

### 4.2 Strain classification and genetic relationships: insights for prevention and vaccine development

Regarding control strategies, understanding the genetic characteristics of the strains is the first step toward developing more effective vaccines and therapeutics. The high percentage similarity observed between the isolated strains and reference strains, such as the 97.1–99.8% for nucleotides and 97.4–99.7% for amino acid sequences in the case of the ORFV011 gene, and the corresponding similarities for the ORFV059 gene, provides valuable information for vaccine design. It suggests that there may be conserved regions within these genes that could be targeted for vaccine development to induce a broad and effective immune response. However, it is also important to consider that genetic variations, even within these relatively similar groups, could potentially affect the efficacy of vaccines. Therefore, continuous monitoring of the genetic changes in ORFV strains is necessary to ensure the long-term effectiveness of control measures.

The phylogenetic tree analysis, with the indication that higher bootstrap values of the evolutionary branches formed by the four strains isolated in Yunnan imply more reliable branch structures and trustworthy genetic distance relationships, further strengthens our understanding of the evolutionary relationships among these strains. This information can be used to identify potential subpopulations or variants within the Yunnan ORFV population that may have different epidemiological or pathogenic characteristics. For example, if a particular branch with a high bootstrap value represents a subgroup of strains that are more virulent or have a different transmission pattern, it would be crucial to focus on these specific strains in control efforts.

#### 4.2.1 *ORFV011* gene

In our study, these ORFV strains were clustered into four groups (Group I–IV) based on the *ORFV011* gene sequences. All our isolated ORFV stains, including YNSLi/2021, YNSLi/2023, YNYLn/2022, YNTJe/2023, were clustered into group I (Figure 4A). Specifically, the *ORFV011* genes in YNSLi/2021 and YNSLi/2023 were more related and clustered with the Fujian SJ1 strain in China, whereas YNYLn/2022 and YNTJe/2023 clustered with the Fujian LYJ strain in China. The *ORFV011* genes in YNSLi/2021 and YNSLi/2023 showed 99.8% homology with the SJ1 strain, while YNYLn/2022 and YNTJe/2023 had the highest homology with the LYJ strain (Table 5).

Future Research Implications: Tracing the movement of animals or animal products that might have carried these strains could provide a more detailed understanding of the transmission pathways. Future studies could use genetic tracing techniques, such as phylogenetic analysis combined with geographical and trade data, to track the origin and spread of these strains. This would help in identifying potential sources of infection and predicting future outbreaks in regions where these related strains are prevalent. Investigating the genetic stability of these strains over time could also be an important area of future research. By monitoring changes in the ORFV011 gene sequence in different generations of the virus, we can better understand how the virus evolves and adapts, which is crucial for developing effective prevention strategies.

Practical Application Implications: The genetic similarities and differences among these strains have significant implications for vaccine development. If a particular strain or group of strains shows a consistent pattern of genetic relatedness, as seen with the clustering based on the ORFV011 gene, it could be a target for vaccine development. However, given the potential for genetic mutations, continuous monitoring of these strains and their genetic changes is essential to ensure vaccine effectiveness. Future vaccine development efforts should focus on creating vaccines that can cover a wide range of related strains, taking into account the genetic variability observed. The close relationship between our isolated strains and specific Fujian strains could also be used to inform regional vaccination strategies. For example, if it is determined that the Fujian SJ1 and LYJ strains are prevalent in a particular region, targeted vaccination campaigns using vaccines designed to protect against these specific strains could be implemented.

#### 4.2.2 *ORFV059* gene

The *ORFV059* gene sequences were also divided into four groups (Groups I–IV) (Figure 4C). YNSLi/2021, YNSLi/2023, YNYLn/2022, and YNTJe/2023 strains were clustered into Group IV based on their *ORFV059* gene sequences, which is currently prevalent in China, India, and Malaysia, represented by the strain OV-SA00. YNSLi/2023 showed close genetic relation to strains from India and Malaysia, while YNSLi/2021, YNYLn/2022, and YNTJe/2023 were more closely related to strains prevalent in China. YNSLi/2023 had the highest homology (99.2%) with strains from Meghalaya and UPM/HSN-20, while YNSLi/2021 had the highest homology (100%) with AH1404. YNYLn/2022 and YNTJe/2023 showed the highest homology with Chinese YX strain (≥99%). The sequence identities and phylogenetic tree analyses indicated that the isolates from Yunnan Province were closely related to ORFV reference strains, particular those from China, such as FJ-SJ1, FJ-2403, LYJ, AH1404, and YX strains (Figures 4A,C). This suggests that regional ORFV strains are prevalent in China, a phenomenon also observed by Zhang et al. (18, 34).

Future Research Implications: Similar to the ORFV011 gene, further exploring the transmission pathways of these strains based on the ORFV059 gene could involve using genetic tracing techniques. By analyzing the genetic relationships between different strains and combining this with geographical and trade data, we can better understand how these strains have spread across different regions. This would help in predicting future outbreaks and identifying potential sources of infection. Studying the functional significance of the genes within the ORFV059 gene group could provide insights into the virus’s pathogenicity and transmissibility. Future research could focus on identifying specific genes within this group that play a key role in these processes, which could then be targeted for therapeutic or prevention strategies.

Practical Application Implications: The identification of strains with high homology, such as YNSLi/2023 with strains from Meghalaya and UPM/HSN-20, and YNSLi/2021 with AH1404, could be used to design more targeted vaccines. However, given the genetic diversity and potential for mutations, continuous monitoring of these strains and their genetic changes is essential to ensure vaccine effectiveness. Future vaccine development efforts should focus on creating vaccines that can cover a wide range of related strains, taking into account the genetic variability observed. The close relationship between our isolated strains and specific Chinese strains could also be used to inform regional vaccination strategies. For example, if it is determined that the AH1404 and YX strains are prevalent in a particular region, targeted vaccination campaigns using vaccines designed to protect against these specific strains could be implemented.

### 4.3 Genetic complexity and strains evolution: challenges and opportunities for prevention and control

We analyzed four reference strains highly homologous to YNSLi/2021, YNSLi/2023, YNTJe/2023, and YNYLn/2022, spanning from 2003 to 2024, including foreign strains from India and Malaysia, and strains from Fujian, Hunan, and Anhui in China (Figures 4A,C). The close affinity of the Yunnan strains with Indian and Malaysian strains suggest cross-regional transmissible, with the highest similarity observed between Yunnan and Fujian, Hunan and Anhui strains. This implies possible transmission from Fujian or intermediary regions, or genetic reassortment during the epidemic. Variations in nucleotide levels between virulent and attenuated strains are predominantly due to single nucleotide mutations (35), which affect physicochemical properties and secondary structure (Tables 7–10). Recent epidemic strains in Yunnan Province show considerable genetic complexity and diversity, complicating ORFV prevention and monitoring. Evolutionary variances in topology suggest that ORFV genes are prone to recombination, potentially resulting in the removal of non-essential genes or the integration of new host genes, leading to robust strain development (21, 36, 37). The ORFV’s mutability and cross-regional transmission also increased the complexity of their geographical distribution. High heterogeneity among ORFV strains from neighboring regions and the presence of multiple strains in the same region without clear correlation between the evolutionary and geographic distance (21, 25, 36) might contribute to recurrent outbreaks (33, 38, 39). Genetic mutations may also accelerate virus transmission and spread, leading to greater differences in pathogenicity and genetic evolution, thus reducing vaccine effectiveness. These results suggest that the evolutionary pathways of *ORFV011* and *ORFV059* gene fragments are complex and diverse. Host immune pressure and environmental factors further influence gene evolution, resulting in more branched and dispersed pathways, related to host adaptability and viral pathogenicity (25, 40). We suspect the 4 isolated ORFV strains may be recombinant viruses. In addition, when assessing the influence of altitude on virus transmission and variation, it is essential to consider a range of factors. Environmental conditions; Vector Dynamics; The distribution of host species; Genetic variation. By employing this multifaceted approach, it is possible to gain insight into the complex interplay between altitude and viral dynamics, which in turn can inform the prediction and management of outbreaks in a range of ecological contexts. The limited sample size of this study precludes further investigation into the impact of altitude on the transmission and variation of ORFV. Accordingly, the altitude data presented in Table 4 are employed solely as sampling markers. To conduct a more comprehensive investigation into the impact of altitude of ORFV, a larger sample size is required.

Future Research Implications: Understanding the mechanisms behind genetic recombination and how it leads to the development of robust strains is crucial. Future research could focus on detailed studies of the genetic recombination process, including the identification of specific genes that are involved in recombination, the conditions that trigger recombination, and the consequences of recombination on the virus’s pathogenicity and transmissibility. This knowledge could be used to develop strategies to prevent or control the emergence of recombinant strains. Investigating the role of host immune pressure and environmental factors in gene evolution in more detail could provide valuable insights. Future studies could focus on how different levels of host immune pressure affect the evolution of ORFV genes, and how environmental factors such as temperature, humidity, and altitude interact with the virus to influence its gene evolution. This knowledge could be used to develop more targeted prevention strategies based on the specific environmental and immune conditions of different regions.

Practical Application Implications: The high genetic complexity and diversity of recent epidemic strains in Yunnan Province pose significant challenges for vaccine selection and application. Future vaccine development efforts should focus on creating vaccines that can cover a wide range of genetic variants, taking into account the genetic variability observed. Additionally, continuous monitoring of the genetic changes of the virus is essential to ensure vaccine effectiveness. Given the complexity of the geographical distribution of ORFV and the potential for recurrent outbreaks, developing more comprehensive outbreak prediction and control models is necessary. These models should incorporate genetic, environmental, and epidemiological factors to better predict and manage outbreaks. For example, by considering the genetic characteristics of different strains, the environmental conditions of different regions, and the movement and trading of livestock, we can more accurately predict where and when outbreaks are likely to occur and take appropriate preventive measures.

### 4.4 Need for systematic prevention and control strategies: building on the findings

The genetic complexity and diversity of recent epidemic strains pose higher demands for vaccine selection and application, potentially leading vaccine failure and difficulties in ORFV prevention and control. The four isolated strains showed low homology with NZ2 and D1701 strains, also indicating epidemiological diversity. These findings highlight the need for systematic and detailed epidemiological and genetic data on ORFV to develop better prevention and control strategies. Considering Yunnan Province’s tropical and subtropical climate, abundant resources, and growing livestock trade (41), it has become a hotspots for ORFV transmission, exacerbating genetic diversity and increasing hazards annually.

Future Research Implications: Conducting larger-scale epidemiological investigations in Yunnan Province and other regions is essential. This could involve collecting more samples from different locations, different host species, and different time periods to capture the full spectrum of the virus’s behavior. By obtaining a more comprehensive understanding of the virus’s distribution, transmission, and evolution, we can develop more targeted prevention strategies. Studying the impact of different factors such as climate, livestock trade, and animal husbandry practices on the transmission and evolution of ORFV in Yunnan Province could provide valuable insights. Future research could focus on how these factors interact with each other and with the virus to influence its behavior. This knowledge could be used to develop more targeted prevention strategies based on the specific conditions of Yunnan Province.

Practical Application Implications: Given Yunnan Province’s specific climate conditions, developing prevention and control strategies that are tailored to these local conditions is necessary. For example, in a tropical and subtropical climate, strategies could focus on measures such as enhancing ventilation in livestock facilities to reduce the humidity and prevent the growth of the virus, and proper management of animal waste to reduce the risk of virus transmission. The identification of regional ORFV strains circulating in China emphasizes the importance of strict inspection of animal products from affected areas to prevent disease spread. Future application efforts should focus on implementing more rigorous quarantine procedures and testing protocols for animal products entering or leaving affected regions. This would ensure that any potentially infected animal products are detected and prevented from spreading the virus.

In summary, determining the precise transmission method of the ORFV strain isolated in Yunnan Province is challenging due to potential introductions via animals imports and product sales. However, regional ORFV strains circulate in China, emphasizing the need for careful examination of animal products from affect areas to prevent disease spread. And this study provides valuable information on ORFV molecular epidemiology and is the first to systematically analyze the molecular characteristics of an ORFV strain isolated from goat farms in Yunnan Province. A systematic epidemiological investigation of ORFV in Yunnan Province is necessary to provide a scientific basis for comprehensive prevention and control of goat stomatitis in the region.

## 5 Conclusion

Through a meticulous examination involving the comparison of the *ORFV011* and *ORFV059* genes, a comprehensive understanding of the intricate ORFV epidemic prevalent in Yunnan Province during the 2021–2023 timeframe was obtained. The results underscored the occurrence of genetic drift and recombination within the epidemic strains found in Yunnan Province, as well as their interplay with strains from different regions, culminating in the emergence of a unique evolutionary lineage. Our isolated ORFV strains are categorized into two distinct gene groups, namely SA00-like and D1701-like. Notably, compared with NZ2 and D1701 strains, these strains exhibit mutations at both nucleotide and amino acid levels, which may lead to immunological failure of the existing ORFV vaccine. Collectively, this study provides a genetic evolutionary analysis. The insights gleaned from this research will contribute significantly to our comprehension of the virus’s transmission pathways and evolutionary processes, thereby establishing a robust scientific basis for the prevention, control, and development of vaccines against ORFV.

## Data Availability

The datasets presented in this study can be found in online repositories. The names of the repository/repositories and accession number(s) can be found in the article/supplementary material.

## Footnotes

^1^ http://staden.sourceforge.net/

^2^ https://blast.ncbi.nlm.nih.gov/Blast

^3^ https://web.expasy.org/protparam/

^4^ https://npsa-pbil.ibcp.fr/
